# The analgesic effect of different interactive modes of virtual reality: A prospective functional near-infrared spectroscopy (fNIRS) study

**DOI:** 10.3389/fnins.2022.1033155

**Published:** 2022-11-15

**Authors:** Xue Deng, Chuyao Jian, Qinglu Yang, Naifu Jiang, Zhaoyin Huang, Shaofeng Zhao

**Affiliations:** ^1^Department of Rehabilitation Medicine, The Eighth Affiliated Hospital of Sun Yat-Sen University, Shenzhen, China; ^2^Key Laboratory of Human-Machine Intelligence-Synergy Systems, Shenzhen Institutes of Advanced Technology, Chinese Academy of Sciences (CAS), Shenzhen, China

**Keywords:** virtual reality, analgesia, functional near infrared spectroscopy, pain, fNIRS

## Abstract

Virtual reality has demonstrated its analgesic effectiveness. However, its optimal interactive mode for pain relief is yet unclear, with rare objective measurements that were performed to explore its neural mechanism.

**Objective:**

This study primarily aimed at investigating the analgesic effect of different VR interactive modes *via* functional near-infrared spectroscopy (fNIRS) and exploring its correlations with the subjectively reported VR experience through a self-rating questionnaire.

**Methods:**

Fifteen healthy volunteers (Age: 21.93 ± 0.59 years, 11 female, 4 male) were enrolled in this prospective study. Three rounds of interactive mode, including active mode, motor imagery (MI) mode, and passive mode, were successively facilitated under consistent noxious electrical stimuli (electrical intensity: 23.67 ± 5.69 mA). Repeated-measures of analysis of variance (ANOVA) was performed to examine its pain relief status and cortical activation, with *post hoc* analysis after Bonferroni correction performed. Spearman's correlation test was conducted to explore the relationship between VR questionnaire (VRQ) items and cortical activation.

**Results:**

A larger analgesic effect on the active (−1.4(95%CI, −2.23 to −0.57), *p* = 0.001) and MI modes (−0.667(95%CI, −1.165 to −0.168), *p* = 0.012) was observed compared to the passive mode in the self–rating pain score, with no significant difference reported between the two modes (−0.733(95%CI, −1.631 to.165), *p* = 0.131), associated with diverse activated cortical region of interest (ROI) in charge of motor and cognitive functions, including the left primary motor cortex (LM1), left dorsal–lateral prefrontal cortex (LDLPFC), left primary somatosensory cortex (LS1), left visual cortex at occipital lobe (LOL), and left premotor cortex (LPMC). On the other hand, significant correlations were found between VRQ items and different cortical ROIs (r = −0.629 to 0.722, *p* < 0.05) as well as its corresponding channels (r = −0.599 to 0.788, *p* < 0.05).

**Conclusion:**

Our findings suggest that VR can be considered as an effective non-invasive approach for pain relief by modulating cortical pain processing. A better analgesic effect can be obtained by exciting and integrating cortical ROIs in charge of motor and cognitive functions. The interactive mode can be easily tailored to be in line with the client's characteristics, in spite of the diverse cortical activation status when an equivalent analgesic effect can be obtained.

## Introduction

Pain is a complex sensory and emotional experience felt by a person in response to a real or an imaginary tissue injury, which can be largely influenced by personal experience and self-perception (Guarin, 2013). Even though there are a variety of approaches for bringing pain relief in a patient, including pharmacological and non-pharmacological types, there is still a worldwide public concern voiced against any inappropriate pain management (Sinatra, 2010). Considering the unsatisfied side effects caused by pharmacological approaches, we recently witnessed an expansion of research on non-pharmaceutical management (Benyamin et al., 2008; Sostres et al., 2010). The updated rationale behind bringing in such a non-pharmaceutical approach is to modulate both personal and environmental factors (Maral and David, 2017). It is suggested that physical exercise can help alleviate pain by exciting the primary motor cortex (M1), thus inhibiting limbic–cortical–thalamic activities, thereby decreasing the negative impact generated by prolonged immobilization on account of sustained pain (Ambrose and Golightly, 2015). On the other hand, studies have suggested that pain perception can be downgraded by orienting visual attention over the body's painful site, as it can strengthen its functional connectivity between the pain network and visual cortex at the occipital lobes (Longo et al., 2012; Karunakaran et al., 2020). It is speculated that a better analgesic effect can be obtained by incorporating these factors for bringing about innovations in current therapeutic approaches.

Even though traditional non-pharmacological management, such as physical exercise and mindfulness breathing, may bear these factors for pain modulation, it is quite challenging to engage a client at a painful status to sustain his/her engagement in a physically/attentively demanding task, letting alone those clients of older age and/or those who are in a severe painful status. Therefore, a tangible approach, which can address this problem, is keenly required. In recent years, virtual reality (VR) has been slowly expanding its application in health care services (Pillai and Mathew, 2019). The rationale behind such an approach like virtual reality is to immerse the client in a three-dimensional (3D) virtual simulated environment, with an entertaining gaming exercise to distract the client's attention from painful stimuli (Triberti et al., 2014; Rizzo and Bouchard, 2019). Over the past years, successive clinical evidences have demonstrated their promising effectiveness for pain reduction, anxiety, and stress management not only in acute pain management caused by a variety of medical procedures, such as wound dressing changes, dental procedures, and peripheral intravenous catheter placement, but also in chronic pain management (Hoffman et al., 2008; Jones et al., 2016; Alshatrat et al., 2018; Gold et al., 2021). However, these findings were mostly accrued *via* subjective ratings with rare objective measurements. In spite of the VR analgesic effect evidenced by a small number of studies using functional magnetic resonance imaging (fMRI), obvious drawbacks of those studies cannot be ignored (Hoffman et al., 2007). For instance, the fMRI as a neuroimaging technique can have its own constraints when observing the neuronal activities during VR interaction, i.e., poor temporal resolution and constrained testing environment (Hennig et al., 2003). On the other hand, even though the distraction hypothesis also suggests that a better analgesic effect can be achieved when a greater extent of attention is required in the VR environment, its optimal interactive mode and the corresponding neural mechanism are both enigmatic (Li et al., 2011; Lier et al., 2020). To overcome the technical constraints mentioned above, functional near-infrared spectroscopy (fNIRS) has been introduced, as it enables the non-invasive quantification of cortical hemodynamics at the near-infrared spectrum (Boas et al., 2004). A recent fNIRS study on VR-induced analgesia clearly discriminated the unique modulation of anterior prefrontal cortex (aPFC) over the premotor cortex in traditional mindful breathing (interoception) by traditional mindful breathing from VR breathing (exteroception), in which the increased visual–auditory cortical activation was associated with diminished functional connection with primary somatosensory cortex (S1) (Hu et al., 2021). It inspired the applicability of fNIRS in VR-induced analgesia studies. Considering its superior temporal resolution and environmental feasibility in comparison with fMRI and electroencephalography (EEG), fNIRS can be used as an optimal neuroimaging tool for observing neuronal activities during VR interaction in an open and unconstrained environment bearing its differential optical properties of hemoglobin (Irani et al., 2007; Yücel et al., 2017).

In the present study, we primarily aimed at investigating the analgesic effect of different VR modes under painful stimuli. Secondarily, we aimed at exploring how cortical pain processing is modulated during different VR interactive modes. Based on the distraction theory, our primary hypothesis suggests that VR with a higher requirement of interactive elements can bring in a better analgesic effect by modulating cortical pain processing.

## Materials and methods

This prospective study was approved by the Medical Ethics Committee of the hospital, with the clinical trial registered at the Chinese Clinical Trial Registry (Ref. No.: ChiCTR2200061536). The study was performed in line with the principle of the Declaration of Helsinki. Written consent form was obtained from all the participants prior to the beginning of the study.

### Participants

Eligible healthy adults aged 18 years or above were enrolled. Participants with disorders enumerated below were excluded: (1) auditory or visual deficit, (2) sensory loss due to peripheral neuropathy or neurological disorders (e.g., peripheral nerve injuries or brain injuries); (3) acute or chronic pain disorders; (4) intake of painkillers or other sensory altering substances (alcohol, etc.) in the recent 2 weeks before the experiment, and (5) motion sickness. Finally, there were 15 young and healthy subjects (4 males, 11 females, age: 21.93 ± 0.59 years) who were recruited.

### Study design and procedures

**Figure 1:**
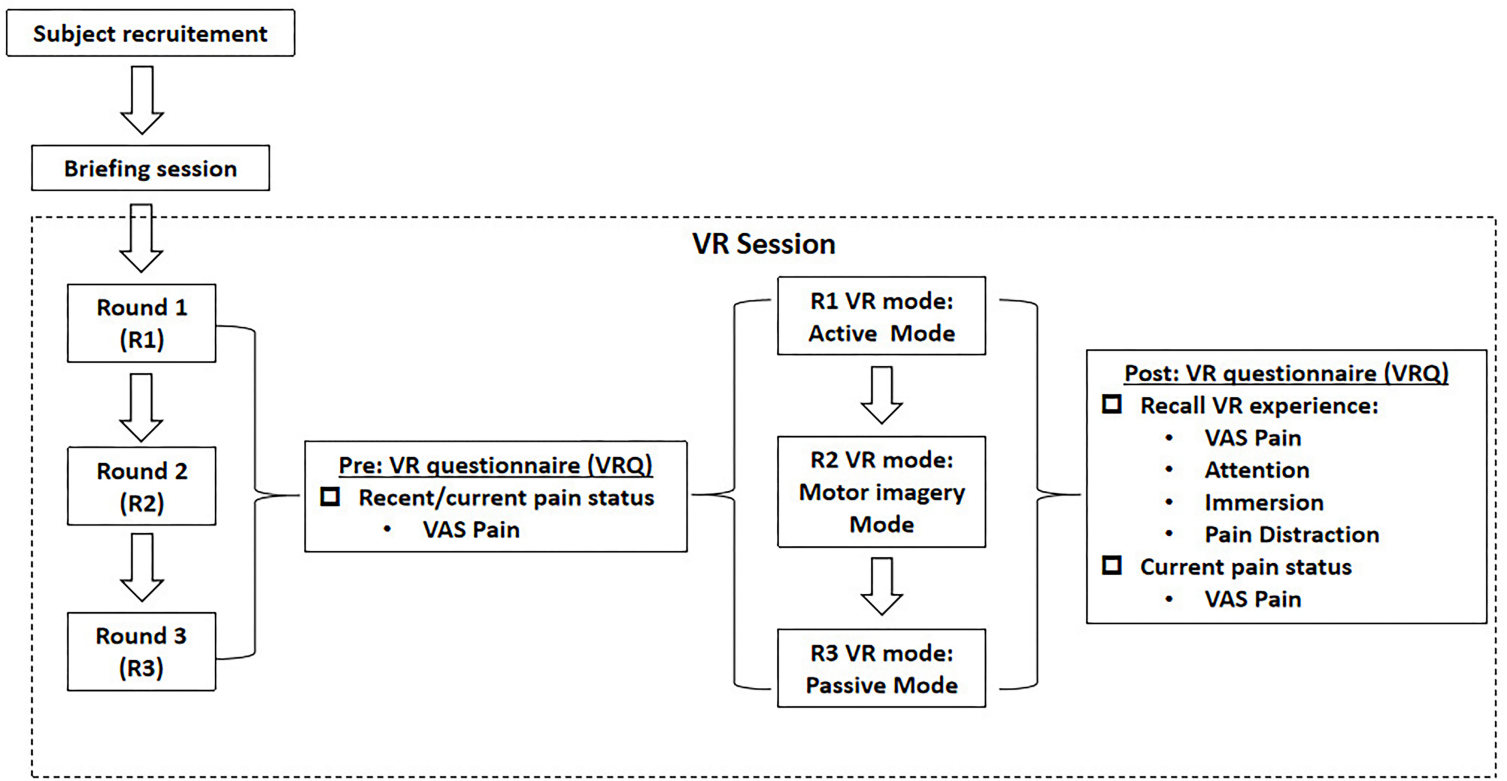
Experimental flow.

As delineated in Figure 1, this study was basically divided into three rounds, with interval between each round of at least 1 day (24 h) to avoid any carryover effect. After the subjects were enrolled by the convenience sampling method, a briefing session was initiated to introduce the experimental flow, to educate on the use of VR device, and to take safety precautions. A VR questionnaire (VRQ) was provided throughout each experimental round. Prior to each VR session, the participants were asked to report their recent pain status *via* the visual-analog scale (VAS) for pain scores in the VRQ, with those who reported any pain excluded. Immediately after the successful completion of the VR session, the participants were asked to recall their subjective VR experience, including the level of pain status, attention, immersion, and pain distraction as well as their current pain status *via* VAS pain scores.

**Figure 2:**
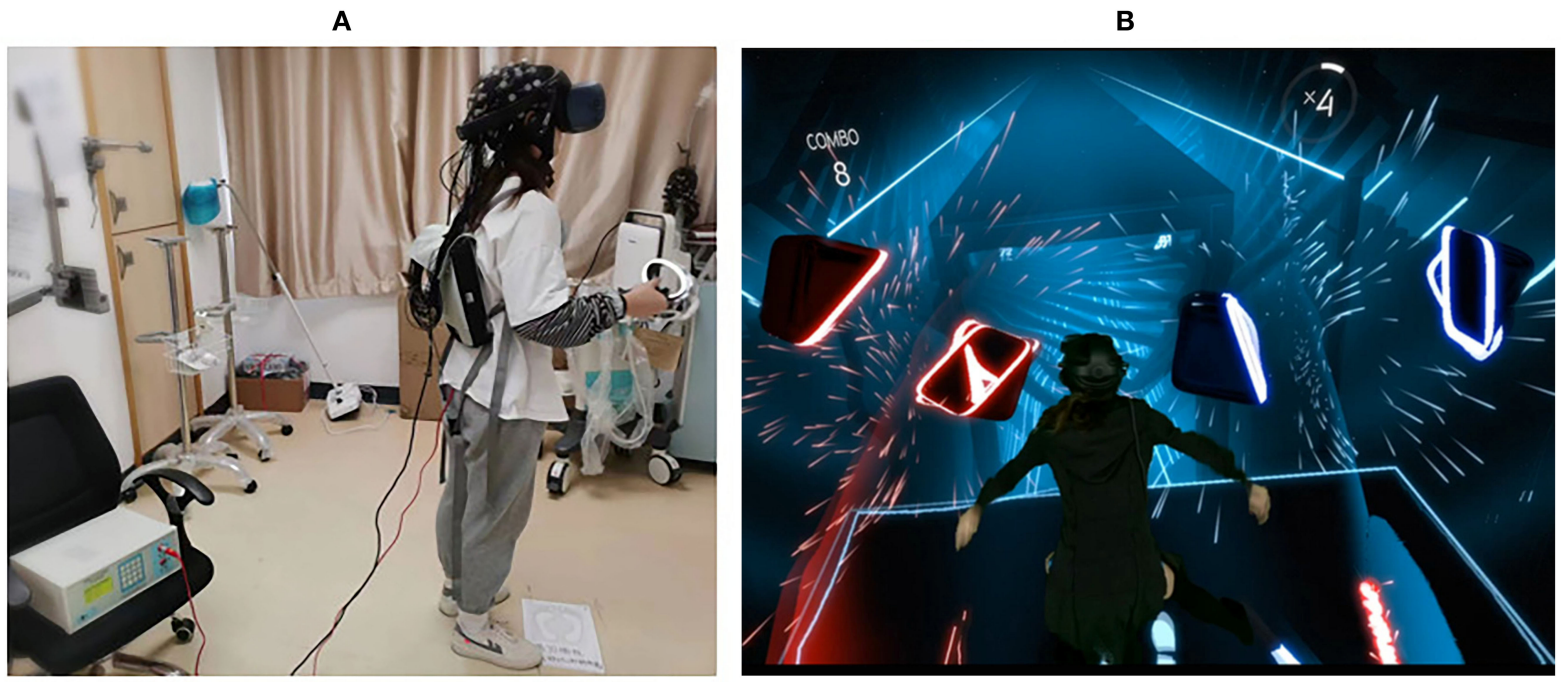
Experimental scenario. **(A)** The scenario of real world; **(B)** the scenario of virtual world (Virtual Scenario of Beat Saber, n.d.).

As shown in Figure 2A, the participant was required to wear a 44-channel fNIRS cap (NirSmart-6000A, Danyang Huichuang Medical Equipment Co. Ltd., China), with a headmounted display (HMD) (HTC VIVE Cosmos, HTC/Valve Co.) placed over his/her head, holding the controllers bilaterally to interact with the VR gaming task. In each round, an electric stimuli equipment (YRKJ-F1002, Yirui Co. Ltd., China) was arranged, with two electrode pads placed on both sides of the participant's lumbar L5/S1 level. The frequency rate was set as 1 Hz, with 1 ms as the frequency width. The intensity of electrical stimuli started from 5 mA upwards, with its intensity fixed when the participant reported that the stimuli were similar to a pinprick sensation (unpleasant, slightly painful). The intensity was constant for every subject throughout each round.

This block study design consists of three parts, including 30-s rest, 190-s VR task, and 60-s recovery. During the 30-s rest period, participants were required to stand still with eyes closed, counting the number in seconds from 1 to 30. In the 190-s task session, subjects were required to interact with the VR gaming task in line with the interactive rules. The last part was a 60-s recovery session. Again, subjects were required to stand still with eyes closed, counting the number in seconds from 1 to 60. The task was conducted under constant electrical stimuli throughout the experiment.

As delineated in Figure 1, the three rounds were composed of active VR mode (R1), motor imagery (MI) mode (R2), and passive VR mode (R3), respectively. A rhythm VR game named <Beat Saber > (Beat Games, Czech) was chosen as the VR task, with the music <pop stars> by K-DA, a virtual K-pop female vocal group that opted for the VR session (Figure 2B). In R1, the participants were required to wield a pair of glowing sabers, slashing a stream of approaching blocks in sync with the song's beats and notes; whereas in R2, the participants were required to track the stream of blocks with their eyes, imaging the correct slashing act without any physical motion; in R3, the participants were required to listen to the music only, with eyes closed and physical motion absent.

### Data analysis

#### fNIRS acquisition

**Figure 3:**
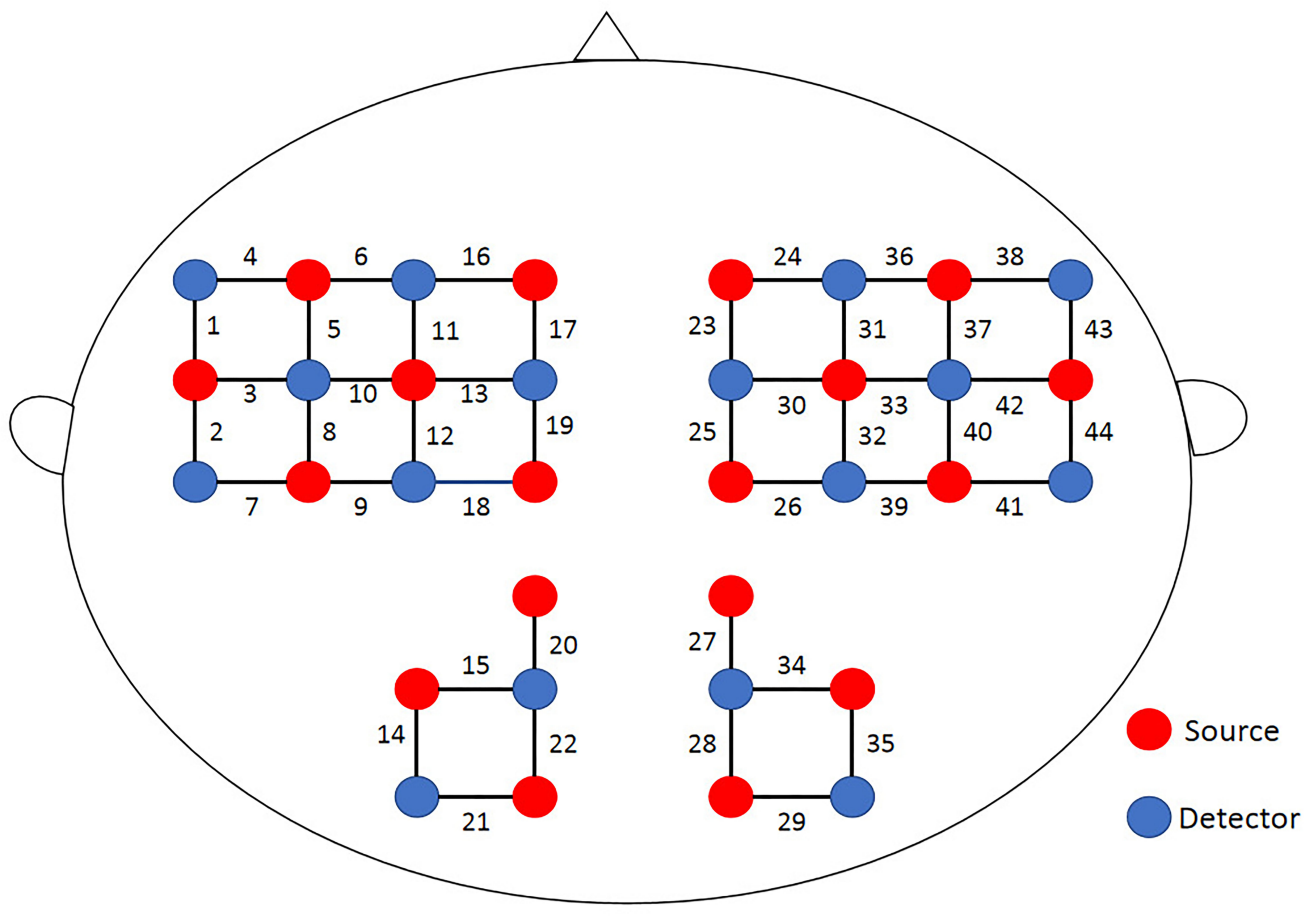
Brain map of channel distribution.

A multichannel portable near-infrared system (NirSmart-6000A, Danyang Huichuang Medical Equipment Co., Ltd., China) was used in this study, taking 11 Hz as the sampling frequency rate with dual near-infrared (near-IR) lights (wavelengths: 730 nm and 850 nm) to detect oxyhemoglobin (HbO) and deoxyhemoglobin (HbR) concentration changes. In accord with the international 10/20 electrode distribution system, the 18 emission sources and 16 detectors (source-detector separation: 3 cm) were arranged over the frontal, parietal, temporal, and occipital regions at both hemispheres, consisting of 44 channels. The spatial locations of sources and detectors were measured by an electromagnetic 3D digitizer device (Patriot, Polhemus, USA) placed on the head of the subject, with acquired coordinates that are converted into coordinates in line with the Montreal Neurological Institute and Hospital (MNI). These coordinates are further projected to the MNI standard brain template using the spatial registration approach in NirSpace (Danyang Huichuang Medical Equipment Co., Ltd., China). A flexible headgear holder was used for reducing signal noise between the emitter and scalp. During the experiment, the excessive light was controlled for better data collection. A brain location map with its distribution of channels is shown in Figure 3.

#### fNIRS data processing

The NIRspark (v1.7.3, Huichuang, China) based on Matlab (v2021a, Natick, USA) was used to analyze the experimental data collected by the fNIRS system. The data processing was performed in the following steps:

① Elimination of the motion artifact: Spline interpolation was taken for data correction. The time window was set as 0.5s. Those signals with any changes beyond 6 standard deviation (std_thr > 6) and 0.5 amplitude (amp_thr >0.5) of the whole time series were considered as the motion artifact (Scholkmann et al., 2010).
② Data conversion: Based on the Beer–Lambert Law, the optical density was converted into HbO and HbR concentrations.
③ Data filter: The raw data were digitally filtered in the bandpass 0.01–0.2 Hz to remove low oscillations, e.g., respiratory and cardiac components.
④ Obtainment of the hemodynamic response function (HRF): The hemodynamic response function's (HRF's) initial time was set to −32 s, and the end time was set to 30 s (with “−32 to −30s” as the reserved baseline state and “−30 to 30s” as the time for a single block paradigm). The HbO concentrations across channels for each subject were superimposed and averaged to compute an average result.
⑤ Calculation of cortical activation: The β value and featured value (FV) were taken as cortical activation in this study. The β values was calculated by employing the Generalized linear model (GLM) as follows:

(1) $$\begin{array}{lll} Y & = & X\beta +\varepsilon ,X\epsilon R^{N\times M},\beta \epsilon R^{M\times L} \end{array}$$

(2) $$\begin{array}{lll} \beta & = & X*Y,X*=\;{\left(X^{T}X\right)}^{-1}X^{T} \end{array}$$

where *X* ϵ *R*^*N*^×*M* denotes the design matrices (where *M* is the number of data points during the recording period and *N* is the number of β dimensions), and β ϵ *R*^*M*^×*L* (where *L* is the number of measurement channels) is the corresponding response signal strength for the HbO parameter. The canonical HRF was chosen as the basic function of GLM. The match between experimental design and HRF values was calculated, then the GLM can derive the β value. The β value represents the intensity of activation triggered by the experimental task in the individual's cerebral cortex. Then, the FV was obtained based on HRF in the rest period (-30 to 0s) being subtracted from that in the task period (0–30 s).

### Statistical analysis

SPSS (v24.0, IBM, USA) was used for statistical analysis. The interactive VR mode from R1 to R3 was considered as the within-subject variable, while the mode sequence was incorporated as the between-subjects variable. The Kolmogorov–Smirnov and Shapiro–Wilk tests were used, revealing data were normally distributed. The obtained data were corrected for multiple comparisons across channels by the false discovery rate (FDR). Repeated-measures analysis of variance (ANOVA) was used to examine the cortical activation (β and FV) and VAS pain scores in the VRQ among the three VR modes. Bonferroni's correction was utilized for multiple comparisons. The confidence level a was defined as 0.05. Finally, Spearman's correlation test was used to examine the relationship between VRQ items and cortical activation (ROIs and corresponding channels).

## Results

### Descriptive characteristics

Finally, there were 15 right-hand dominant participants (number of males/females: 4/11; age: 21.93 ± 0.59 years) who were enrolled, with no one dropped out throughout the experiment. The overall mean intensity of electrical stimuli was 23.67 ± 5.69 mA, equivalent of VAS 4/10 subjectively reported by the enrolled participants during the resting period, with no one reporting any side effect after the experiment. The VAS scores during VR task from R1 to R3 were 2.33/10 (active mode), 3.07/10 (MI mode), and 3.73/10 (passive mode) respectively (Table 1).

**Table 1:** Descriptive characteristics.

**Demographics**					
N		15			
Age (year): Mean ± SD		21.93 ± 0.59			
Gender (Male/Female, N)		4/11			
Hand dominance(Left/Right)		0/15			
Electric stimulus intensity (mA): mean ± SD		23.67 ± 5.69			
VRQ		Pain	Attention	Immersion	Pain Distraction
R1	Pre VR task	4/10	-	-	-
	During VR task	2.33 ± 1.23/10	7.87 ± 2.42	8.73 ± 1.39	6.53 ± 3.36
	Post VR task	0	-	-	-
R2	Pre VR task	4/10	-	-	-
	During VR task	3.07 ± 0.7/10	6.2 ± 2.11	6.53 ± 2.29	6.2 ± 2.27
	Post VR task	0	-	-	-
R3	Pre VR task	4/10	-	-	-
	During VR task	3.73 ± 0.88/10	4.93 ± 2.19	4.87 ± 2.67	4.73 ± 2.4
	Post VR task	0	-	-	-

VRQ, VR questionnaire; R1, active mode R2, motor imagery; R3, passive mode.

### VR-induced analgesic effect during different VR interactive modes

**Figure 4:**
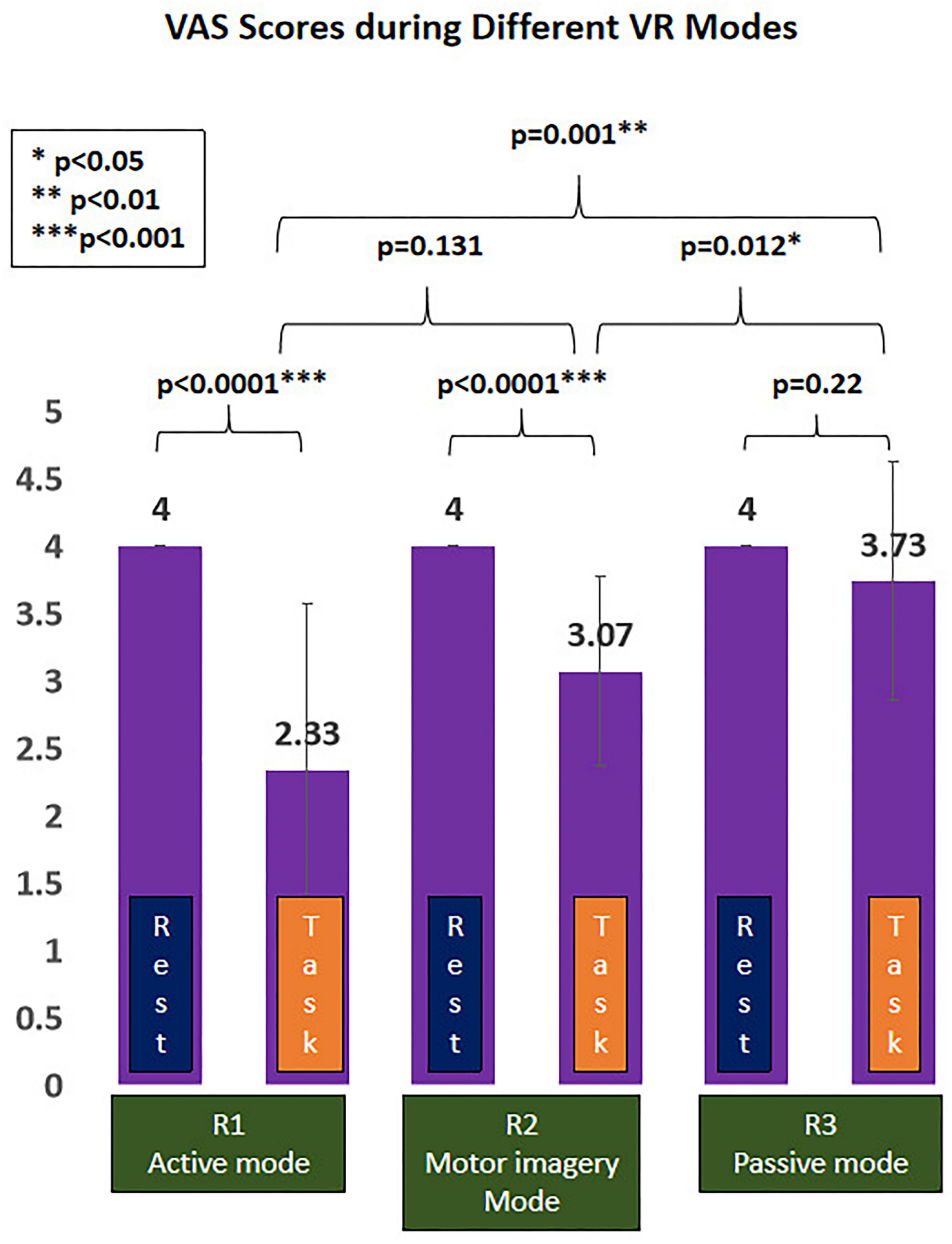
Virtual reality (VR)-induced analgesic effect during different VR interactive modes. *p < 0.05; **p < 0.01; ***p < 0.001.

The painful status during different modes of VR interaction is presented in Figure 4. A significant effect of VR analgesia was found in the subjectively reported pain scores (F(1.743, 24.401)=11.47, *p* < 0.0001). *Post hoc* analysis revealed significant difference between R1 (active mode) and R3 (passive mode) (−1.4 (95%CI, −2.23 to −0.57), *p* = 0.001), R2 (MI mode), and R3 (passive mode) (−0.667 (95%CI, −1.165 to −0.168), *p* = 0.012), whereas no significant difference was found between R1(active mode) and R2 (MI mode) (−0.733 (95%CI, −1.631 to 0.165), *p* = 0.131).

### Cortical activation during different VR interactive modes

**Figure 5:**
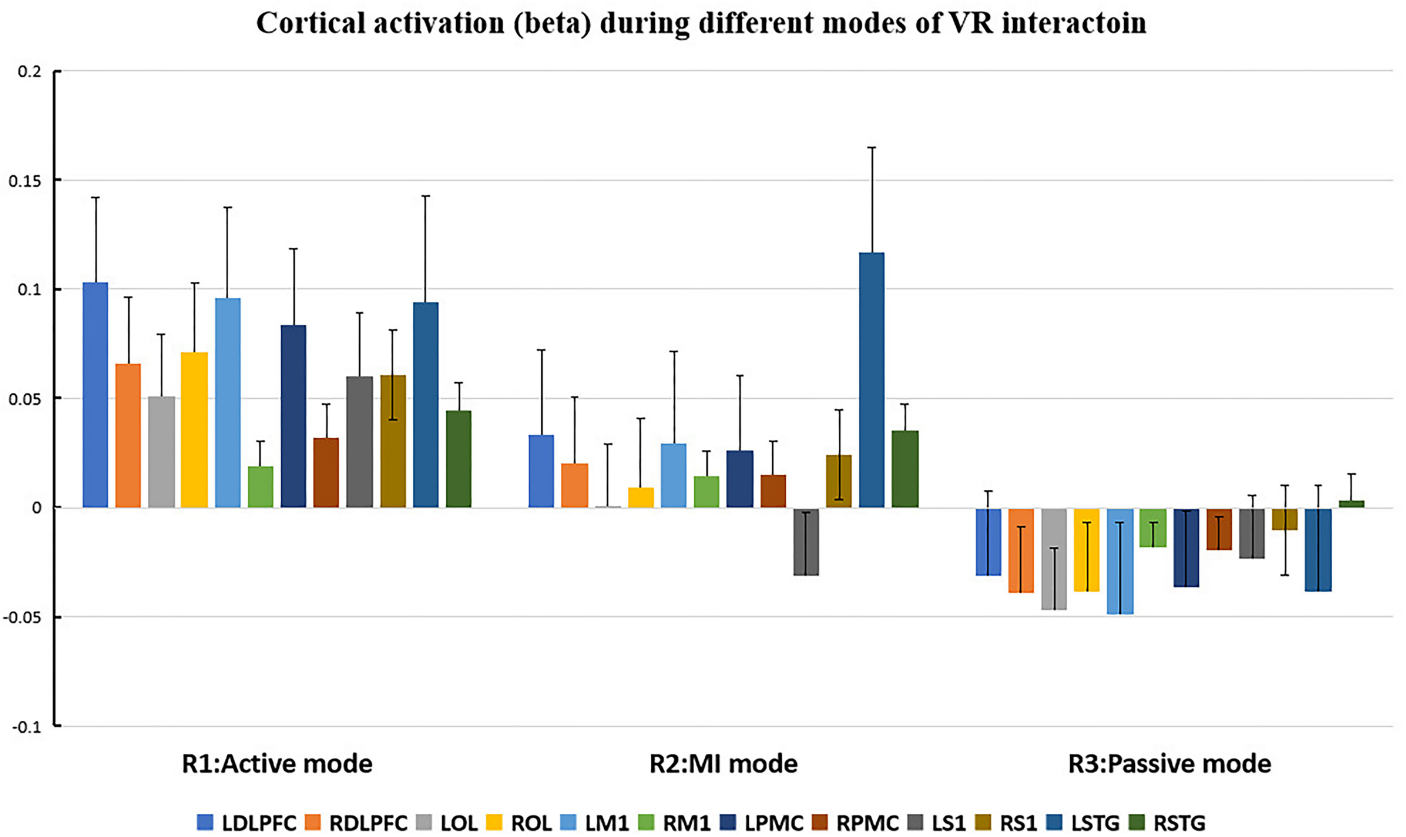
Cortical activation (β value) during different modes of virtual reality (VR) interaction. LDLPFC, left dorsal–lateral prefrontal cortex; RDLPFC: right dorsal–lateral prefrontal cortex; LOL, left occipital lobe; ROL, right occipital lobe; LM1, left primary motor cortex; RM1, right primary motor cortex; LPMC, left premotor cortex; RPMC, right premotor cortex; LS1, left primary somatosensory cortex; RS1, right primary somatosensory cortex; LSTG, left superior temporal gyrus; RSTG, right superior temporal gyrus.

**Figure 6:**
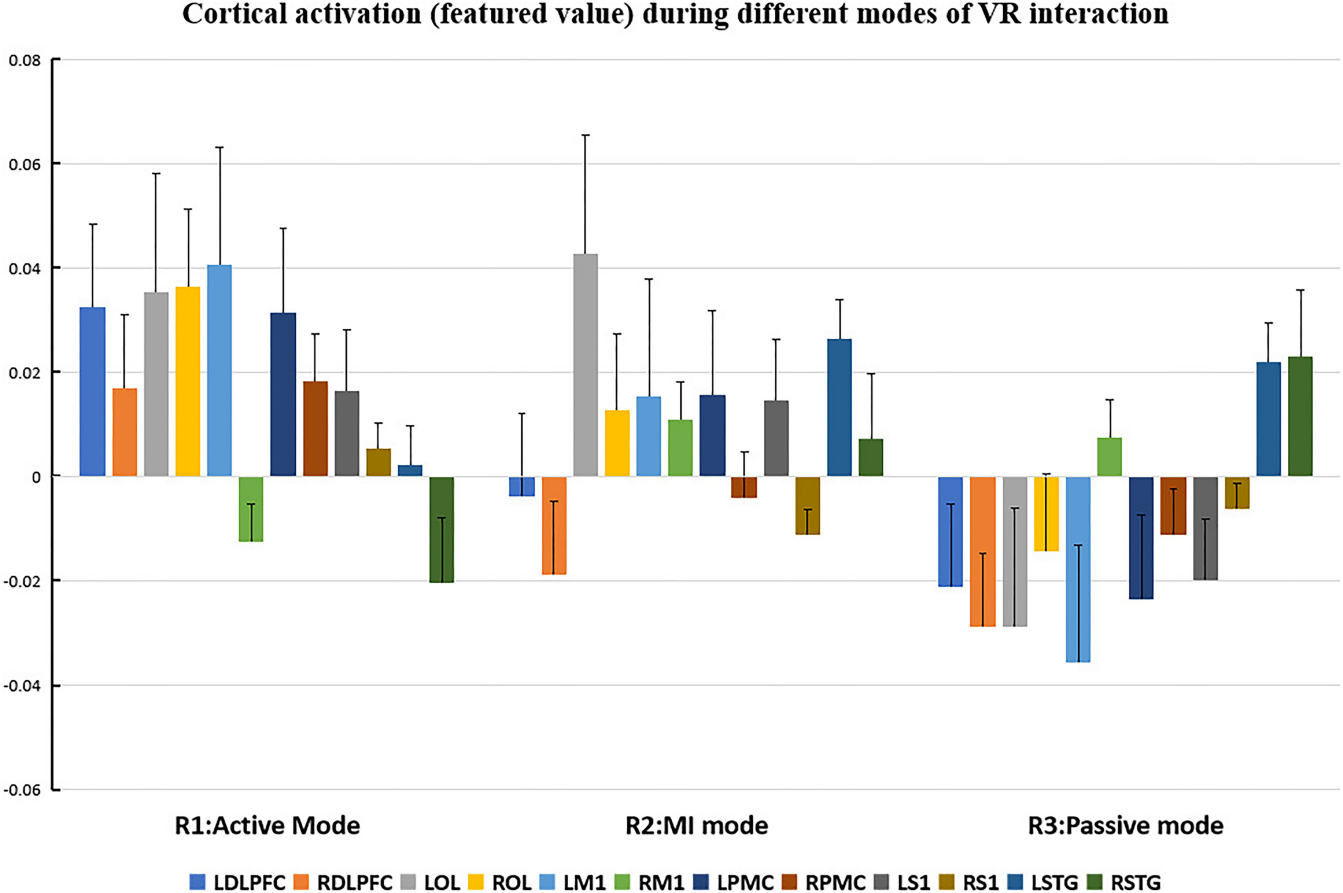
Cortical activation (featured value) during different modes of virtual reality (VR) interaction. LDLPFC: left dorsal–lateral prefrontal cortex; RDLPFC, right dorsal–lateral prefrontal cortex; LOL, left occipital lobe; ROL, right occipital lobe; LM1, left primary motor cortex; RM1, right primary motor cortex; LPMC, left premotor cortex; RPMC, right premotor cortex; LS1, left primary somatosensory cortex; RS1, right primary somatosensory cortex; LSTG, left superior temporal gyrus; RSTG: right superior temporal gyrus.

The cortical regions of interest (ROIs) and their corresponding channels, associated with the MNI coordinates, are described in Table 2. The obtained β and featured values (FVs) in each ROI, including dorsal–lateral prefrontal cortex (DLPFC), primary motor cortex (M1), premotor cortex (PMC), primary somatosensory (S1), superior temporal gyrus (STG), and occipital lobe (OL) in both hemispheres, were also delineated, with the numerical analysis for each ROI plotted in Figures 5, 6 correspondingly.

**Table 2:** Cortical activation [β and featured values (FVs)] during different virtual reality (VR) interactive modes.

**ROI**	**BA**	**Anatomic label**	**CH**	**MNI coordinates**			**Beta**			**Feature value**		
				**X**	**Y**	**Z**	**R1**	**R2**	**R3**	**R1**	**R2**	**R3**
LDLPFC	9	Dorsolateral prefrontal cortex	16	12.45	−3.15	15.75	0.10 ± 0.13	0.03 ± 0.13	−0.03 ± 0.11	0.03 ± 0.07	0.00 ± 0.02	−0.02 ± 0.07
RDLPFC	9	Dorsolateral prefrontal cortex	24	14.7	−4.65	6.8	0.07 ± 0.14	0.02 ± 0.14	−0.04 ± 0.07	0.02 ± 0.07	−0.02 ± 0.08	−0.03 ± 0.05
LOL	18	V2	14	1.25	−9.2	14.55	0.05 ± 0.09	0.00 ± 0.11	−0.05 ± 0.10	0.04 ± 0.05	0.04 ± 0.05	−0.03 ± 0.07
	17,18,19	V1, V2, V3	15	3.2	−10.9	14.45						
	19	V3	20	5.65	−12.05	14.05						
	18	V2	21	0.85	−9.7	13.6						
	17	V1	22	2.8	−11.4	13.5						
ROL	19	V3	27	7.2	−13.35	8.45	0.07 ± 0.07	0.01 ± 0.07	−0.04 ± 0.09	0.04 ± 0.04	0.01 ± 0.05	−0.01 ± 0.06
	17	V1	28	4.25	−12.85	7.7						
	17	V1	29	2.75	−11.65	6.05						
	18	V2	34	5.4	−12.	6.75						
	17,18,19	V1,V2,V3	35	3.9	−11.6	5.1						
LM1	4	Primary motor cortex	13	11.3	−6.1	16.15	0.1 ± 0.11	0.03 ± 0.12	−0.05 ± 0.11	0.04 ± 0.05	0.02 ± 0.06	−0.04 ± 0.06
	4	Primary motor cortex	19	11.6	−8.2	15.25						
RM1	4	Primary motor cortex	25	13.45	−9.3	8.4	0.02 ± 0.1	0.01 ± 0.09	−0.02 ± 0.15	−0.01 ± 0.08	0.01 ± 0.04	0.01 ± 0.10
	4	Primary motor cortex	30	13.7	−7.7	6.75						
LPMC	6	Pre-motor and supplementary motor cortex	11	10.8	−3.9	16.8	0.08 ± 0.09	0.03 ± 0.14	−0.04 ± 0.08	0.03 ± 0.04	0.02 ± 0.06	−0.02 ± 0.06
	6	Pre-motor and supplementary motor cortex	17	12.95	−5.35	15.1						
RPMC	6	Pre-motor and supplementary motor cortex	23	14.75	−6.45	8.3	0.03 ± 0.07	0.02 ± 0.08	−0.02 ± 0.12	0.02 ± 0.06	0.00 ± 0.06	−0.01 ± 0.07
	6	Pre-motor and supplementary motor cortex	31	13.65	−5.9	5.25						
LS1	2	Primary somatosensory cortex	8	6.7	−5.1	17.25	0.06 ± 0.05	−0.03 ± 0.11	−0.02 ± 0.08	0.02 ± 0.05	0.01 ± 0.04	−0.02 ± 0.05
	1, 2, 3	Primary somatosensory cortex	12	9.3	−6.8	16.95						
RS1	1	Primary somatosensory cortex	32	12.3	−8.8	5.35	0.06 ± 0.11	0.02 ± 0.11	−0.01 ± 0.14	0.01 ± 0.07	−0.01 ± 0.06	−0.01 ± 0.07
	2	Primary somatosensory cortex	40	10.1	−7.45	3.25						
LSTG	22	Superior temporal gyrus	3	6.15	−3.05	17	0.09 ± 0.14	0.12 ± 0.11	−0.04 ± 0.18	0.00 ± 0.04	0.03 ± 0.08	0.02 ± 0.04
RSTG	22	Superior temporal gyrus	42	9.45	−5.	2.7	0.04 ± 0.08	0.04 ± 0.1	0.00 ± 0.1	−0.02 ± 0.08	0.01 ± 0.06	0.02 ± 0.05

ROI, Region of interest; BA, Broadmann area; CH, channel; MNI, Montreal Neurological Institute and Hospital (MNI); R1, active mode R2, motor imagery; R3, passive mode; LDLPFC, left dorsal lateral prefropntal cortex; RDLPFC, right dorsal lateral prefrontal cortex; LOL, left occipital lobe; ROL, right occipital lobe; LM1, left primary motor cortex; RM1, right primary motor cortex; LPMC, left premotor cortex; RPMC, right premotor cortex; LS1, left primary somatosensory cortex; RS1, right primary somatosensory cortex; LSTG, left superior temporal gyrus; RSTG, right superior temporal gyrus;V1, primary visual cortex, V2, visual association cortex 2; V3, visual association cortex 3.

Repeated measures (RM)-ANOVA revealed significant difference in β values of LM1 (F(6.37, 20.3), *p* = 0.009), LS1 (F(3.708, 24.628), *p* = 0.041), and LOL (F(7.973, 22.5), *p* = 0.003) as well as the FV in LPMC (F(8.379, 21.1), *p* = 0.002), LM1 (F(4.356, 22.627), *p* = 0.027), and LOL (F(9.249, 20.725), *p* = 0.002). On the other hand, in terms of the individual channel, it revealed a significant difference in the β values of channel 13 (F(3.963, 15.938), *p* = 0.04) and channel 15 (F(11.274, 14.917), *p* = 0.002) as well as FV in channel 11 (F(5.255, 18.478), *p* = 0.017), channel 17 (F(4.349, 13.85), *p* = 0.035), and channel 19 (F(5.782,23.117), *p* = 0.01).

**Figure 7:**
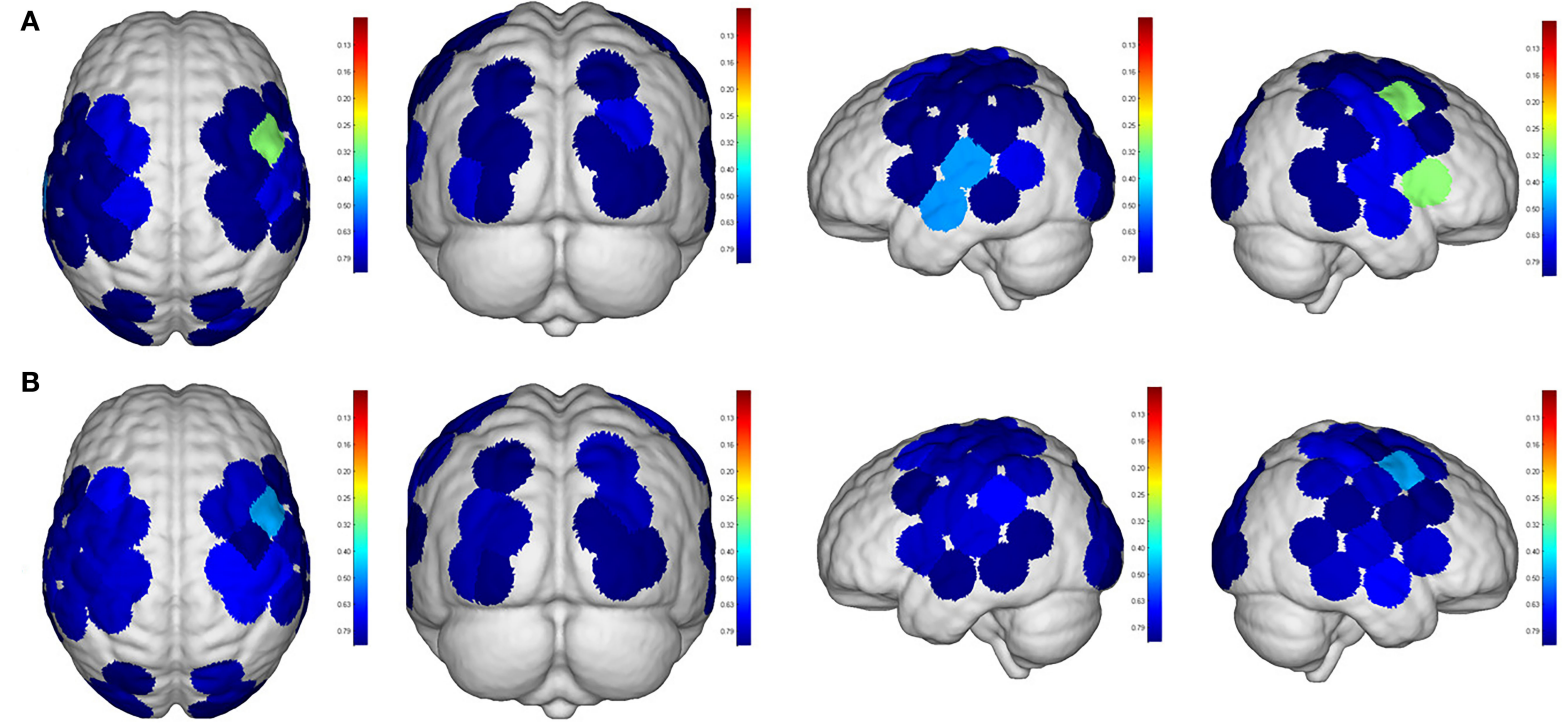
Comparisons of cortical activation between R1 and R2. R1: Active mode; R2: motor imagery (MI) mode; **(A)**: β value; **(B)**: featured value; color bar: 0.1 to 0.9.

**Figure 8:**
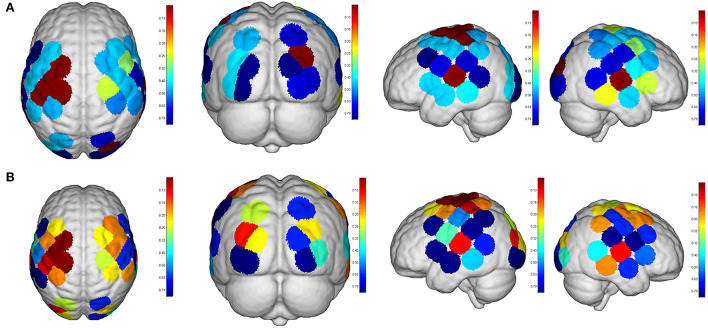
Comparisons of cortical activation between R1 and R3. R1: active mode; R3: passive mode; **(A)**: β value; **(B)**: featured value; color bar: 0.1 to 0.9.

**Figure 9:**
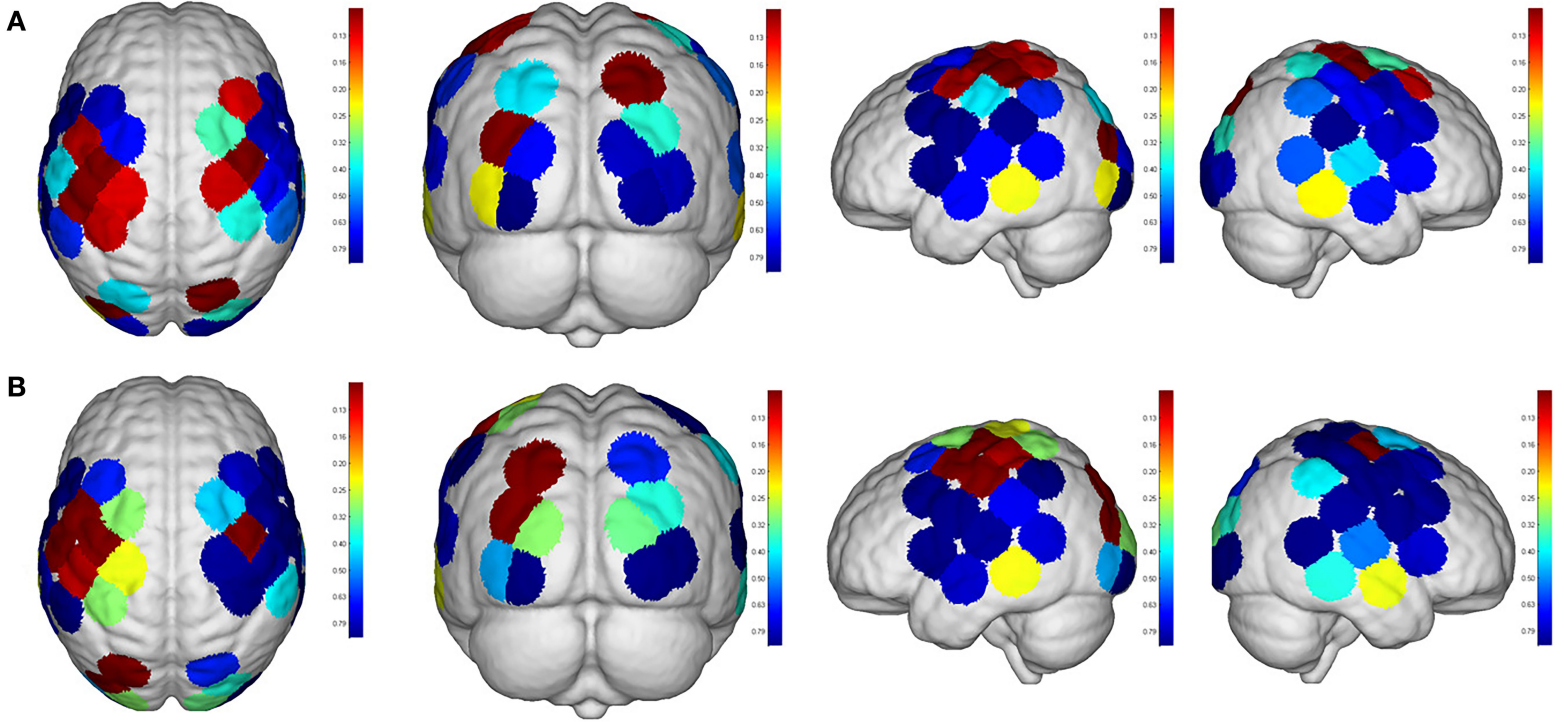
Comparisons of cortical activation between R2 and R3. R2: motor imagery (MI) mode; R3: passive mode; **(A)**: β value; **(B)**: featured value; color bar: 0.1 to 0.9.

Even though no significant difference was found in the cortical activation of ROIs between R1 and R2 (Figure 7), there were significant differences of ROIs in comparisons with ROIs and individual channels for R1 and R3 (Figure 8) as well as R2 and R3 (Figure 9). Regarding β values, *post hoc* analysis after Bonferroni correction revealed significant difference between R1 and R3 in channel 13 (0.133(95%CI, 0.028 to 0.238), *p* = 0.012), channel 15 (0.14(95%CI, 0.028 to 0.252), channel 16 (0.025(95%CI, −0.087 to 0.138), *p* = 0.017), and channel 17 (0.17(0.037, 0.304), *p* = 0.013) (Figure 8A); R2 and R3 in LOL (0.103 (95%CI, 0.041 to 0.166) *p* = 0.003) and channel 15 (0.17(95%CI, 0.037 to 0.304), *p* = 0.013) (Figure 9A).

Regarding FV, *post hoc* analysis after Bonferroni correction revealed significant difference between R1 and R3 in channel 11 (0.091(95%CI, 0.004 to 0.177), *p* = 0.014), channel 17 (0.071(95%CI, −0.003 to 0.146), *p* = 0.016), and channel 19 (0.083(95%CI, 0.011 to 0.135), *p* = 0.022) as well as LPMC (0.069(95%CI, 0.027 to.11), *p* = 0.003) (Figure 8B); R2 and R3 in LOL (0.067(95%CI, 0.032 to 0.101), *p* = 0.004) (Figure 9B).

### Correlation between VRQ items and cortical roi/channel activation

Regarding correlations between VRQ items and β values, significant correlations were found between attention and LM1 (r = 0.609, *p* = 0.016) as well as its corresponding channel 19 (r = 0.677, *p* = 0.006), pain and right S1 [right primary somatosensory cortex (RS1)] (r = −0.588, *p* = 0.021), pain distraction and right PMC (RPMC) (r = −0.528, *p* = 0.043) as well as RDLPFC (r = −0.668, *p* = 0.009) in R1; significant correlation was found between pain distraction and RDLPFC in R2 (r = 0.531, *p* = 0.042); significant correlations were found between attention and RDLPFC (r = 0.587, *p* = 0.027), attention and RS1 (r=0.543, *p* = 0.045) as well as between immersion and RS1 (r = 0.539, *p* = 0.047) in R3 (Table 3).

**Table 3:** Spearman's correlation between virtual reality questionnaire (VRQ) items and cortical activation (β and featured values).

		**LDLPFC**	**RDLPFC**	**LOL**	**ROL**	**LM1**	**RM1**	**LPMC**	**RPMC**	**LS1**	**RS1**	**LSTG**	**RSTG**
Beta value	P_R1	-	-	-	-	-	-	-	-	-	−0.588*	-	-
	A_R1	-	-		-	0.609* (Ch19:0.677**)	-	-	-	-	-	-	-
	I_R1	-	-	-	-	-	-	-	-	-	-	-	-
	PD_R1	-	0.668**	-	-	-	-	-	−0.528*	-	-	-	-
	P_R2	-	-	-	-	-	-	-	-	-	-	-	-
	A_R2	-	-	-	-	-	-	-	-	-	-	-	-
	I_R2	-	-	-	-	-	-	-	-	-	-	-	-
	PD_R2	-	0.531*	-	-	-	-	-	-	-	-	-	-
	P_R3	-	-	-	-	-	-	-	-	-	-	-	-
	A_R3	-	-	-	-	-	-	-	-	-	0.543*	-	-
	I_R3	-	-	-	-	-	-	-	-	-	0.539*	-	-
	PD_R3	-	-	-	-	-	-	-	-	-	-	-	-
Featured Value	P_R1	-	-	-	-	-	-	-	-	-		-	-
	A_R1	-	-	-	-	0.772** (Ch19:0.788***)	-	-	-	-	0.543*	-	-
	I_R1	-	-	-	-	-	-	-	-	-	-	-	-
	PD_R1	-	-	-	-	-	-	-	-	-	-	-	-
	P_R2	-	-	-	-	-	-	0.539*	-	-	-	-	-
	A_R2	-	-	0.557*	-	-	-	-	−0.62* (Ch31:−0.599*)	-	-	-	−0.629*
	I_R2	-	0.574*	-	-	-	-	-	-	-	-	-	-
	PD_R2	-	-	-	-	-	-	-	-	-	-	-	-
	P_R3	-	-	-	-	-	-	-	-	-	-	-	-
	A_R3	-	0.538*	-	-	-	-	-	-	-	-	-	-
	I_R3	-	-	-	-	-	-	-	-	-	-	-	-
	PD_R3	-	0.743**	-	-	-	-	-	-	-	-	-	-

* *p* < 0.05; **p < 0.01; ***p < 0.001.

VRQ, VR questionnaire; R1, active mode; R2, motor imagery mode; R3, passive mode, P, Pain, A, attention; I, immersion; PD, pain distraction; LDLPFC, left dorsal lateral prefropntal cortex; RDLPFC, right dorsal lateral prefrontal cortex; LOL, left occipital lobe; ROL, right occipital lobe; LM1, left primary motor cortex; RM1, right primary motor cortex; LPMC, left premotor cortex; RPMC, right premotor cortex; LS1, left primary somatosensory cortex; RS1, right primary somatosensory cortex; LSTG, left superior temporal gyrus; RSTG, right superior temporal gyrus.

On the other hand, regarding correlations between VRQ items and featured values (FVs), a significant correlation was found between attention and LM1 (r = 0.772, *p* = 0.001) associated with its corresponding channel 19 (r = 0.788, *p* < 0.001), as well as RS1 (r = 0.543, *p* = 0.036) in R1; immersion and RDLPFC (r = 0.574, *p* = 0.032) in R2; pain and LPMC (r = 0.539, *p* = 0.047), attention and RPMC (r=-0.62, *p* = 0.014) associated with its corresponding channel 33(r=-0.599, *p* = 0.022), LOL (r = 0.557, *p* = 0.039) as well as RSTG (r = −0.629, *p* = 0.016) in R2; correlation was found between attention and RDLPFC (r = 0.538, *p* = 0.047) as well as pain distraction and RDLPFC (r = 7.43, *p* = 0.002) in R3 (Table 3).

## Discussion

To our knowledge, this is the first study which has investigated the effectiveness of different VR interactive modes for pain relief through fNIRS. In terms of the outcome generated through both subjective and objective measurements, we aimed at exploring VR as an analgesic by observing the cortical pain processing during painful stimuli. Throughout each round, the VR context and the electrical stimuli were both consistent, while the interactive mode was particularly designed in terms of its engaged level. According to the distraction theory, it is suggested that not only the interactive element of VR but also the level of attention is paid to the VR environment, which may contribute to the ultimate analgesic effect (Mccaul and Malott, 1985; Gutierrez-Maldonado et al., 2011). Our finding demonstrated a better analgesic effect can be achieved to be associated with higher attention, immersion, and pain distraction during interaction with the VR context, which was consistent with a prior VR study using EEG, suggesting a better analgesic effect in the active VR mode is associated with reduced amplitudes of N1 and P3 (Lier et al., 2020). In addition, another finding in our studies revealed no significant difference between active and MI mode, implicating equivalent analgesic effect can be attainable when a sufficient level of attention was distracted in the VR context. This finding can be inspiring for those clients who tend to be quite immobile at painful status, as a similar analgesic effect can be achieved when a less active mode can be used.

Pain was processed, based on the sensory stimuli and behavior status by integrating different cortical information from various ROIs (Karunakaran et al., 2020). Therefore, the diverse analgesic effect associated with varied activated cortical area during different VR modes can be indicative of an altered pain perceptual processing. In our experiment, since the intensity of the electrical stimulus was never changed for every subject, the noxious input can be constant from peripheral to the spinal level. In this way, the area that modulates noxious stimuli by VR can only be situated at the thalamal–cortical area, where the correspondent cortical ROIs in charge of motor and cognitive function were involved.

As for the motor-relevant cortical area, significant difference in the activation in LM1 between active and passive mode was found (Figure 8). In addition, a dual positive correlation between attention and LM1 activation, associated with its corresponding channel in the active mode, was also specified (Table 3). M1 was basically in charge of motor planning, initiation, and execution of voluntary movement by processing cortical information from parietal, frontal, and temporal cortical regions (Wei-Ju et al., 2015). Since the corticospinal activation can be inhibited by acute painful stimuli, this ROI has been a critical target for pain relief by exciting it through invasive or non-invasive approaches (Svensson et al., 2003; Lopes et al., 2019). In our experiment, the active VR mode requires the participant's visual attention to identify and track the fast-moving blocks, plan the dimensions of movement, i.e., direction, speed, etc., to initiate and finally execute the slashing act in an appropriate way. It can be analogous to a process of non-invasive stimulation (exercise) to excite the primary motor cortex, meanwhile distracting his/her attention to interact in the VR context. Whereas in the passive VR mode, the level of attention was much less needed, in which the M1 was least activated compared to the other modes (Figures 5, 6). Therefore, M1 can be considered as a critically targeted area in a VR-induced analgesic approach.

Regarding cognition-relevant cortical area, a similar finding was observed in the DLPFC, in which its activation in the active VR mode was higher compared to the less engaged VR modes, bearing a strong correlation with cognitive factors during different modes of VR interaction (Table 3, Figures 5, 6). The DLPFC (Brodmann Area 9) is considered to be relevant to higher-order cognitive processing related to attention, working memory, and inhibition of responses (Karunakaran et al., 2020). Previous fMRI studies reported its role in pain processing, including detection, perception, and suppression of pain (Apkarian et al., 2004). It was found that non-invasive stimulation of the LDLPFC appears to exhibit an anti-nociceptive effect, thus increasing pain tolerance (Brighina et al., 2011). In our study, a higher activation of DLPFC was found in the active VR mode compared to the less engaged modes (Figures 5, 6). Therefore, it can be considered as a non-invasive approach to excite the DLPFC, thus sustaining participant's attention to the VR task during noxious stimuli.

Even though the role of visual cortex at OL in pain processing has not been established, Huff et al. (2022) suggested that visual cortex is critical for the consious perception of visual stimuli, visual-guided attention, and motor action. Previous studies have reported the use of fNIRS in observing the function of visual cortex for attention and working memory following mild traumatic brain injury. However, there is yet not any study using fNIRS to evaluate pain-associated changes in the visual cortex (Takahashi et al., 2000). In our experiment, it was found that the visual cortex was correlated with attention (Table 3), with higher increased activation in the active VR mode compared to those less engaged modes, associated with better pain relief status (Figures 4, 5). Even though no casual relationship was found between visual cortex and pain relief in our study, there were animal studies that reported atrophy in visual cortex may occur following intense stress, which is reportedly similar to human beings in painful status (Yoshii et al., 2017).

Similar to visual cortex, a higher activation of PMC was also found in the active mode in comparison with those less engaged VR modes (Figures 4, 5), associated with better analgesic effect. This frontal cortical region is part of Brodmann Area 6 in charge of movement preparation (Chouinard and Paus, 2006). The PMC function in motor activities for planning, imagination, and control of movement was evidenced by previous fNIRS studies but no specific studies relevant to pain processing were reported (Pawan et al., 2017). Nevertheless, PMC was suggested in planning the escape when facing an aversive event such as pain (Haines and Mihailoff, 2018). In this way, a higher level of VR interaction can be considered as a way of better “escaping” from the pain status, which may help to explain the consistent activation.

Nevertheless, several drawbacks cannot be ignored in our study. The participants were all young and healthy, such that the promising analgesic effect can be biased. Regarding the combined use of VR headset and fNIRS equipment, the weight of HMD headset as well as the optode of the fNIRS cap over the scalp may generate some pain. The HbO collected by fNIRS was not nociceptive specific but reflected an overall response following the noxious stimulation. The head movement may vary in different interactive modes, resulting in various motion artifacts. To reduce the impact, a time window (1–2 min) for adaptation was provided immediately after placing these devices on the head before the experiment. To minimize the possible data interference, a prevalently used data-processing method, which enables semi-automatic detection and reduction of movement artifacts, was taken based on moving standard deviation and spline interpolation (Scholkmann et al., 2010). In addition, multiple channels in the correspondent ROIs were averaged among subjects, which can represent the repeated measures of analyzed ROIs in the time-locked phase. It is believed that a more robust result can be obtainable when the future studies bearing a larger sample size with broader spectrum such as age, gender, and specific pain-related disorder are made accountable.

## Conclusion

Conclusively, our findings suggest that the VR mode with a higher level of engagement can bring in a better analgesic effect by modulating motor and cognitive cortical ROIs in charge of pain processing. They further suggest that the VR interactive mode can be easily tailored to be in line with the client's status when the equivalent analgesic effect can be attainable. Our findings have contributed to suggest VR as a non-pharmacological analgesic method for pain management.

## Data availability statement

The raw data supporting the conclusions of this article will be made available by the authors, without undue reservation.

## Ethics statement

The studies involving human participants were reviewed and approved by Medical Ethics Committee of the Eighth Affiliated Hospital of Sun Yat-Sen University. The patients/participants provided their written informed consent to participate in this study.

## Author contributions

XD contributed to study design, data acquisition, and drafting and revision of manuscript. SZ contributed to study concept and methodology design. CJ contributed to data processing. QY and NJ contributed to data analysis. ZH contributed to data collection and data management. All authors contributed to the article and approved the submitted version.

## Funding

This study was sponsored by National Natural Science Foundation of China (Ref. No. 82001356), Shenzhen Science and Technology Program (Ref. No. JCYJ20190808102001750, JCYJ20210324115014038, and JCYJ20220818102016034), and Futian Healthcare Research Project (Ref. No. FTWS2021089).

## Conflict of interest

The authors declare that the research was conducted in the absence of any commercial or financial relationships that could be construed as a potential conflict of interest.

## Publisher's note

All claims expressed in this article are solely those of the authors and do not necessarily represent those of their affiliated organizations, or those of the publisher, the editors and the reviewers. Any product that may be evaluated in this article, or claim that may be made by its manufacturer, is not guaranteed or endorsed by the publisher.
